# Supplementary material for: Bot fit: A novel approach to assessing lower limb muscular strength
Source: PLoS One. 2026 Mar 26;21(3):e0339187. doi: 10.1371/journal.pone.0339187 (PMC13021169; doi:10.1371/journal.pone.0339187)
Supplement: Attachment — Submitted filename: Response to Reviewers_revised_Final_110625.docx [file pone.0339187.s005.docx]

**Dear Editor and Reviewers:**

Our previous paper, [PONE-D-25-09036] - [EMID:834ef8ac76b1d107], entitled " Bot Fit: A Novel Approach to Assessing Lower Limb Muscular Strength," has been revised in response to your valuable comments. We appreciate your insightful feedback and have made the following modifications accordingly.

We would like to express our sincere gratitude once again for your invaluable comments and suggestions.

Sincerely,

DaeEun Kim

Biological Cybernetics Lab

School of Electrical & Electronic Engineering

Yonsei University

Sinchon, Seodaemoon-gu

Seoul, 120-749

South Korea

**Journal Requirements:**

**When submitting your revision, we need you to address these additional requirements.**

**1. Please ensure that your manuscript meets PLOS ONE's style requirements, including those for file naming. The PLOS ONE style templates can be found at**

**https://journals.plos.org/plosone/s/file?id=wjVg/PLOSOne_formatting_sample_main_body.pdf and**

[**https://journals.plos.org/plosone/s/file?id=ba62/PLOSOne_formatting_sample_title_authors_affiliations.pdf**](https://journals.plos.org/plosone/s/file?id=ba62/PLOSOne_formatting_sample_title_authors_affiliations.pdf)

**We thank the editor for the guidance regarding PLOS ONE’s formatting requirements. As suggested, we have carefully revised the manuscript to ensure it aligns with the journal’s style guidelines. Specifically, we corrected formatting elements such as removing periods after figure and table labels (e.g., changing “Fig.” to “Fig” and ensuring consistency in table references). We have also reviewed file naming conventions and updated all supplementary materials accordingly.**

**Please let us know if any additional formatting adjustments are required.**

**2. We note that the grant information you provided in the ‘Funding Information’ and ‘Financial Disclosure’ sections do not match.

When you resubmit, please ensure that you provide the correct grant numbers for the awards you received for your study in the ‘Funding Information’ section.**

**Thank you for pointing out the inconsistency between the ‘Funding Information’ and ‘Financial Disclosure’ sections. As suggested, we have carefully reviewed and revised both sections to ensure that the grant numbers and funding sources are fully aligned and accurately reported. The corrected and consistent grant information is now reflected in the resubmitted manuscript.**

**3. Thank you for stating the following financial disclosure:
“This study was supported by Samsung Electronics, Republic of Korea. This work was supported by the National Research Foundation of Korea (NRF) grant funded by the Korea government (MSIT) (Grant No. NRF-2020S1A5A2A03044672, 2020R1A2B5B01002395).”**

**Please state what role the funders took in the study. If the funders had no role, please state: "The funders had no role in study design, data collection and analysis, decision to publish, or preparation of the manuscript."
If this statement is not correct you must amend it as needed.
Please include this amended Role of Funder statement in your cover letter; we will change the online submission form on your behalf.**

**Thank you for your guidance regarding the funding disclosure. As requested, we have added a statement clarifying the role of the funders both in the cover letter and in the manuscript.**

**Cover Letter**

***Funding Statement:*** *This study was supported by Samsung Electronics, Republic of Korea. This work was also funded by the National Research Foundation of Korea (NRF) through a grant from the Korean government (MSIT) (Grant No. 2020R1A2B5B01002395), and by the Korea Institute for Advancement of Technology (KIAT) through a grant from the Korean government (MOTIE) (Grant No. P0020535, The Competency Development Program for Industry Specialists).*

***Role of Funders:*** *The funders had no role in study design, data collection and analysis, decision to publish, or preparation of the manuscript.*

**Manuscript – Funding Statement Section:**
*The authors declare that financial support was received for the research, authorship, and/or publication of this article. This study was supported by Samsung Electronics, Republic of Korea. Additional support was provided by the National Research Foundation of Korea (NRF), funded by the Korean government (MSIT) (Grant No. 2020R1A2B5B01002395), and by the Korea Institute for Advancement of Technology (KIAT), funded by the Korean government (MOTIE) (Grant No. P0020535, The Competency Development Program for Industry Specialists).
The funders had no role in study design, data collection and analysis, decision to publish, or preparation of the manuscript.*

**4. Thank you for stating the following in the Acknowledgments Section of your manuscript:
“This study was supported by Samsung Electronics, Republic of Korea. This work was supported by the National Research Foundation of Korea (NRF) grant funded by the Korea government (MSIT) (Grant No. NRF-2020S1A5A2A03044672, 2020R1A2B5B01002395).”**

**We note that you have provided funding information that is not currently declared in your Funding Statement. However, funding information should not appear in the Acknowledgments section or other areas of your manuscript. We will only publish funding information present in the Funding Statement section of the online submission form.
Please remove any funding-related text from the manuscript and let us know how you would like to update your Funding Statement. Currently, your Funding Statement reads as follows:
“The author(s) received no specific funding for this work”

Please include your amended statements within your cover letter; we will change the online submission form on your behalf.**

**Thank you for pointing this out. In response to your comment, we have removed all funding-related information from the Acknowledgments section and other parts of the manuscript. Instead, we have incorporated the appropriate funding information exclusively into the Funding Statement section.**

**The revised Funding Statement now reads as follows:**

***Funding Statement*** *The author(s) declare that financial support was received for the research, authorship, and/or publication of this article. This study was supported by Samsung Electronics, Republic of Korea. Additional support was provided by the National Research Foundation of Korea (NRF), funded by the Korean government (MSIT) (Grant No. 2020R1A2B5B01002395), and by the Korea Institute for Advancement of Technology (KIAT), funded by the Korean government (MOTIE) (Grant No. P0020535, The Competency Development Program for Industry Specialists).
The funders had no role in study design, data collection and analysis, decision to publish, or preparation of the manuscript.*

**We confirm that this revised Funding Statement is included in both the cover letter and the manuscript, and that all other mentions of funding have been removed as per the journal’s guidelines.**

**5. In the online submission form, you indicated that “The datasets used and analyzed in the manuscript are available from the corresponding author upon reasonable request (Email:
kbmang@yonsei.ac.kr, daeeun@yonsei.ac.kr). The dataset includes personal information such as age, height, weight, and blood pressure; therefore, access to the data is restricted to ensure the privacy and confidentiality of participants.”.**

**All PLOS journals now require all data underlying the findings described in their manuscript to be freely available to other researchers, either 1. In a public repository, 2. Within the manuscript itself, or 3. Uploaded as supplementary information.**

**This policy applies to all data except where public deposition would breach compliance with the protocol approved by your research ethics board. If your data cannot be made publicly available for ethical or legal reasons (e.g., public availability would compromise patient privacy), please explain your reasons on resubmission and your exemption request will be escalated for approval.**

**We thank the editors for highlighting the importance of data availability. In response, we have revised our data availability plan to fully comply with *PLOS ONE*’s open data policies while maintaining adherence to ethical and contractual obligations.**

**We now provide four anonymized datasets as Supporting Information:**

- **muscle_param.csv: Contains muscle strength parameters used in our regression analysis, with all personally identifiable information (e.g., age, height, weight, blood pressure) removed in accordance with Section 10 of our IRB-approved protocol.**
- **demographics.csv: Includes participants’ general demographic information (age, height, weight, and BMI) without any identifiers.**
- **angle_data.zip: Includes raw joint angle data (left and right legs) collected from 25 participants (sub1 to sub25) during each exercise protocol. These data reflect movement characteristics only and contain no sensitive or identifiable information.**
- **kneeup_correction.csv: Contains processed knee-up exercise data used to generate Figure 6 in the manuscript.**

**However, we are unable to share the full raw dataset generated by the Bot Fit system, which includes proprietary motor signals, internal performance computations, and signal processing algorithms, due to confidentiality agreements with Samsung Electronics. The Bot Fit system remains under active development, and these data contain commercially sensitive information.**

**We are prepared to provide supporting documentation, including email correspondence with Samsung Electronics, to verify these restrictions. Therefore, we respectfully request an exemption from full data disclosure based on these ethical and contractual constraints.**

**We believe that the anonymized datasets provided as Supporting Information offer sufficient transparency and reproducibility for the scientific community.**

**Additionally, the Data Availability Statement in the manuscript has been revised as follows:**

**Data Availability Statement**
Anonymized datasets sufficient to replicate the analyses and findings reported in the manuscript are provided as Supporting Information:

(1) muscle_param.csv, containing muscle strength parameters used in the regression analysis;

(2) demographics.csv, containing participant age, height, weight, and BMI;

(3) angle_data.zip, including raw joint angle data (left and right legs) from all exercise protocols; and

(4) kneeup_correction.csv, containing processed knee-up exercise data underlying Figure 6.

Researchers with justified purposes may contact the corresponding author for general inquiries about the study.

However, all data requests must be directed to a non-author data contact:

Minchul Kim, Samsung Electronics Co., Ltd.

Email: min1chul.kim@samsung.com

This contact will review and handle data requests in accordance with Samsung Electronics’ confidentiality and intellectual property policies.

**6. Please include captions for your Supporting Information files at the end of your manuscript, and update any in-text citations to match accordingly. Please see our Supporting Information guidelines for more information:** [**http://journals.plos.org/plosone/s/supporting-information**](http://journals.plos.org/plosone/s/supporting-information)**.**

**We thank the editors for this helpful instruction regarding Supporting Information. In response, we have added clear captions for the Supporting Information files at the end of the manuscript, in accordance with the journal’s guidelines.**

**The following files have been included:**

***S1 Dataset.*** *muscle_param.csv. Anonymized dataset of muscle strength parameters for 25 participants used in the statistical analyses. All personally identifiable information has been removed in accordance with IRB guidelines.*

***S2 Dataset.*** *demographics.csv. Anonymized demographic information (age, height, weight, BMI) for 25 participants, corresponding to Table1 in the manuscript.*

***S3 Dataset.*** *angle_data.zip. Compressed folder containing left and right leg joint angle data for each exercise protocol across 25 participants (sub1–sub25). These raw signals represent lower-limb motion captured by the Bot Fit system during resistance exercise trials.*

***S4 Dataset****. kneeup_correction.csv. Processed knee-up exercise data underlying Figure6, including raw and corrected repetitions and angle measurements.*

**We have also updated the in-text citations throughout the manuscript to appropriately reference these Supporting Information files.**

**7. Please review your reference list to ensure that it is complete and correct. If you have cited papers that have been retracted, please include the rationale for doing so in the manuscript text, or remove these references and replace them with relevant current references. Any changes to the reference list should be mentioned in the rebuttal letter that accompanies your revised manuscript. If you need to cite a retracted article, indicate the article’s retracted status in the References list and also include a citation and full reference for the retraction notice.**

**Thank you for your comment. We have carefully reviewed our reference list to ensure its completeness and accuracy. We confirm that none of the references cited in our manuscript have been retracted, and therefore no changes to the reference list were necessary.**

**If any issues regarding the cited literature arise during the editorial process, we will gladly address them without delay.**

**Reviewers' comments:

Reviewer's Responses to Questions**

**Comments to the Author**

**1. Is the manuscript technically sound, and do the data support the conclusions?

The manuscript must describe a technically sound piece of scientific research with data that supports the conclusions. Experiments must have been conducted rigorously, with appropriate controls, replication, and sample sizes. The conclusions must be drawn appropriately based on the data presented.**

**Reviewer #1: Yes**

**Reviewer #2: Yes**

**We sincerely thank Reviewer #1 and Reviewer #2 for their positive evaluations regarding the technical soundness of our study and the strength of the evidence supporting our conclusions. We appreciate your recognition of the rigor in our experimental design, including appropriate controls, replication, and sample size. Your feedback reinforces our confidence in the validity and relevance of our findings.**

**2. Has the statistical analysis been performed appropriately and rigorously?**

**Reviewer #1: Yes**

**Reviewer #2: Yes**

**We sincerely thank Reviewer #1 and Reviewer #2 for recognizing the rigor and appropriateness of our statistical analyses. We carefully selected methods such as correlation analysis, regression modeling, and cluster validation in accordance with our study objectives and data characteristics. Your acknowledgment reinforces the reliability and validity of our analytical approach.**

**3. Have the authors made all data underlying the findings in their manuscript fully available?

The** [**PLOS Data policy**](http://www.plosone.org/static/policies.action#sharing) **requires authors to make all data underlying the findings described in their manuscript fully available without restriction, with rare exception (please refer to the Data Availability Statement in the manuscript PDF file). The data should be provided as part of the manuscript or its supporting information, or deposited to a public repository. For example, in addition to summary statistics, the data points behind means, medians and variance measures should be available. If there are restrictions on publicly sharing data—e.g. participant privacy or use of data from a third party—those must be specified.**

**Reviewer #1: No**

**Reviewer #2: Yes**

**We thank the reviewers for highlighting the importance of data availability. In response to Reviewer #1’s concern, we have revised our data availability plan to fully comply with the *PLOS ONE* Data Availability policy while maintaining adherence to ethical and contractual obligations.**

**We now provide four anonymized datasets as Supporting Information:**

- **muscle_param.csv: Contains muscle strength parameters used in the regression analysis, with all personally identifiable information (e.g., age, height, weight, blood pressure) removed in accordance with Section 10 of our IRB-approved protocol.**
- **demographics.csv: Includes participants’ demographic data (age, height, weight, and BMI) in de-identified form.**
- **angle_data.zip: Contains raw joint angle data (left and right legs) from all exercise protocols, reflecting movement characteristics only and excluding any sensitive or personally identifiable information.**
- **kneeup_correction.csv: Contains processed knee-up exercise data used for the analyses presented in Figure 6.**

**Due to the proprietary nature of the Bot Fit system, which remains under development in collaboration with Samsung Electronics, the full raw dataset—including internal motor signals, performance computations, and signal processing algorithms—cannot be publicly released. These data contain commercially sensitive information protected under confidentiality agreements.**

**To verify these restrictions, we are prepared to submit supporting documentation, including correspondence with Samsung Electronics. Accordingly, we respectfully request an exemption from full data disclosure on the grounds of ethical and contractual constraints.**

**We believe that the anonymized datasets provided offer sufficient transparency and reproducibility for the scientific community. In addition, we have designated a non-author data contact (Minchul Kim, Samsung Electronics; *min1chul.kim@samsung.com*) to handle data access requests in accordance with Samsung Electronics’ confidentiality and intellectual property policies.**

**4. Is the manuscript presented in an intelligible fashion and written in standard English?

PLOS ONE does not copyedit accepted manuscripts, so the language in submitted articles must be clear, correct, and unambiguous. Any typographical or grammatical errors should be corrected at revision, so please note any specific errors here.**

**Reviewer #1: Yes**

**Reviewer #2: Yes**

**We sincerely thank both Reviewer #1 and Reviewer #2 for their positive comments regarding the clarity and language quality of our manuscript. We are pleased that the manuscript was found to be intelligible and well-written. In preparing the revised version, we carefully rechecked the text and corrected minor typographical and grammatical errors to further improve clarity and readability.**

**5. Review Comments to the Author

Please use the space provided to explain your answers to the questions above. You may also include additional comments for the author, including concerns about dual publication, research ethics, or publication ethics. (Please upload your review as an attachment if it exceeds 20,000 characters)**

**Reviewer #1: Dear Authors,

Thank you for the opportunity to review your manuscript. Your study addresses a timely and relevant topic in the fields of biomechanics and physical performance assessment, offering an innovative perspective on wearable technology for strength evaluation. Overall, your manuscript is well-structured, methodologically sound, and clearly written. Your experimental design is solid, with clear objectives, appropriate controls, and well-defined procedures. The sample size, while modest, is adequate for the exploratory nature of the study, and ethical approval is appropriately addressed. The statistical approach is thorough and well-executed. You employed appropriate tests (e.g., Pearson correlation, regression modeling, HCA) and supported your findings with effect sizes and power analysis, which strengthens the credibility of your results. Including accuracy metrics and classification performance for cluster analysis was a notable strength. The manuscript is written in clear, standard English. The structure flows logically, and the scientific language is appropriate for an academic audience. Only minor stylistic refinements may be necessary at revision for polishing, but these do not hinder readability. You have transparently acknowledged the main limitations (e.g., sample size, variation in technique), and your conclusions are well-supported by the presented data. However, according to PLOS ONE’s policies, data must be fully and publicly available. Currently, the manuscript states that data are available upon request, due to privacy concerns. I suggest that you anonymize the dataset (removing identifiable features) and deposit it in a public repository. Alternatively, provide a formal justification from your IRB or ethics committee explaining the restriction.**
**We sincerely thank Reviewer #1 for the thorough and encouraging review. We are grateful for your recognition of the study’s novelty, methodological rigor, and overall clarity.**

**In response to your comment regarding data availability, we have revised the manuscript and now provide anonymized datasets as Supporting Information in accordance with PLOS ONE’s data policy and our IRB protocol. Specifically, we have included:**

***S1 File (muscle_param.csv):* A de-identified dataset of muscle strength parameters used in our regression analyses.**

***S2 File (demographics.csv):* Anonymized participant demographic data (age, height, weight, BMI) corresponding to Table 1.**

***S3 File (angle_data.zip):* Joint angle data (left and right leg) by exercise protocol for 25 participants. This dataset excludes all personally identifiable information.**

***S4 File kneeup_correction.csv):* Processed knee-up exercise data underlying Figure~6, including raw and corrected repetitions and angle measurements.**

**These files are cited directly in the manuscript text as Supporting Information, ensuring compliance with the journal’s data policy and facilitating transparency and reproducibility.**

**As requested, these files are cited directly in the manuscript text:**

***Page 7, Lines 210–212:*** *“We derived muscle parameters by analyzing records obtained from the physical information and exercise equipment of subjects on Day 1 of the experimental protocols to assess the base muscular strength of all subjects (Table 1, S1 File and S2 File).”*

***Page 8, Lines 228–233:*** *“As shown in Fig D, the similarity between the time intervals derived from motor signals and sEMG signals exceeds 0.8, indicating strong temporal alignment. Building on this result, we developed performance metrics based on motor signal analysis for all exercise outcomes. Using the data provided in S3 File, the number of exercise repetitions can be accurately extracted by leveraging various internal motor signals from the robot.”*

***Page 10, Lines 326–329:***

*“Performing the exercise with a smaller angle makes it relatively easier to perform more repetitions, but performing it at the correct angle allows for a more accurate assessment of the subject’s strength as provided in S4 File.”*

**Due to contractual constraints with Samsung Electronics, the full raw dataset—including proprietary signal processing and internal algorithms—cannot be publicly released. However, all essential variables used in our analysis are included in the Supporting Information to ensure transparency and reproducibility. Additional documentation (e.g., confirmation emails) is available upon request to verify the limitations on full data sharing.**

**We respectfully request that this approach be considered sufficient to meet the journal’s data availability requirements.**

**Minor Suggestions for Improvement:

Consider including a brief discussion on the potential application of Bot Fit in clinical or rehabilitation contexts. Clarify whether the Bot Fit system could be adapted to populations with mobility impairments, given the emphasis on healthy young adults. In summary, this is a well-conducted study with methodological rigor and practical relevance. With minor revisions — particularly regarding data availability — I believe the manuscript is suitable for publication.**

**Sincerely,
Rodrigo Oliveira**

**We sincerely thank the reviewer for the thoughtful suggestions and encouraging evaluation of our manuscript. In response to the comment regarding the potential application of Bot Fit in clinical or rehabilitation contexts, we have added a dedicated paragraph in the revised Discussion section. This addition highlights the potential of Bot Fit to support individuals with mobility impairments or those undergoing physical therapy, thereby broadening its applicability beyond healthy young adults.**

***Page 13, Lines 427–449:***

*Despite the promising findings, this study has several limitations. First, it was conducted with 25 participants, which limits the generalizability of the results. Second, the relatively short exercise durations call for further research to validate the approach over longer training periods. Additionally, variability in exercise angles and individual participant conditions necessitates clearer standardization to ensure consistent exercise form and control for daily physical fluctuations.*

*One critical factor influencing knee-up performance was the exercise angle, as shown in Fig 6. Because the knee-up exercise was performed at a high cadence (132 bpm), a lower knee angle made it easier to maintain speed. To ensure consistency and accurate assessment [55], we monitored movement patterns using Bot Fit’s motor signals and direct observation. Participants maintained a knee-up angle of less than 65 degrees, which enabled controlled and reproducible performance.*

*Beyond its application to strength assessment in healthy adults, the Bot Fit system may also hold promise for clinical and rehabilitation settings. Although this study focused on young, healthy individuals, the wearable robotic platform could potentially be adapted to support individuals with reduced mobility or those undergoing physical therapy. Future studies involving older adults or clinical populations will be necessary to evaluate the system’s usability and effectiveness in these contexts.*

*Nevertheless, this study introduces an innovative approach to muscular strength assessment using the hip-joint exoskeleton robot, Bot Fit. By addressing the limitations of traditional strength assessment methods, our findings underscore the potential of wearable robotics to enable dynamic, real-time, and accessible evaluation of muscular performance across various settings.*

**In line with this addition, we have also revised the *Conclusion* section to emphasize the broader clinical relevance and versatility of Bot Fit. These revisions reflect the reviewer’s insightful recommendation and enhance the practical implications of our work.**

***Page 14, Lines 451–472:***

*Assessing muscular strength is fundamental to overall well-being and exercise performance. This study introduced Bot Fit, a hip-joint exoskeleton robot, as an innovative tool for evaluating muscular strength in healthy individuals through safe and controlled resistance exercises, with automated performance analysis based on motor signals.*

*A total of 25 participants assessed their baseline muscular strength and performed five resistance exercises using Bot Fit. The analysis revealed strong correlations between muscular strength and exercise performance, particularly in squats, lunges, and knee-ups. Notably, complex movements such as squats and lunges were associated with short-duration, high-intensity performance, whereas simpler exercises like knee-ups reflected sustained performance at high speeds. These findings indicate that Bot Fit offers a precise, reproducible, and convenient method for strength assessment, eliminating the need for additional equipment or human supervision.*

*Beyond general fitness applications, Bot Fit may also hold potential in clinical and rehabilitation settings, particularly for individuals with limited mobility. With further validation and refinement, the system could support broader use across different populations and health conditions.*

*Future research should expand the sample size and incorporate additional data sources, such as sEMG and motor signal analysis, to improve model validity and robustness. Through continued development, Bot Fit may serve as a valuable platform for both fitness evaluation and rehabilitation support, contributing to more efficient and accessible muscular strength assessment.*

**Reviewer #2: It is a valuable paper, edited in a clear and easy-to-follow style. Iconografy facilitates the understanding of the paper. The results are valuable, as it proves the efficacy of a innovative tool in evaluating muscular strength, which might be useful for rehabilitation.**

**We sincerely thank Reviewer #2 for the encouraging and positive feedback. We are pleased to hear that you found the manuscript clearly presented and that the visual elements enhanced the understanding of our findings.**

**We also appreciate your comment on the potential utility of Bot Fit in rehabilitation contexts. In response to this, and to a similar suggestion from Reviewer #1, we have added a brief discussion in the Discussion section to emphasize the potential for Bot Fit to support individuals with reduced mobility or those undergoing physical therapy (Page 13, Lines 438–448). We have also updated the Conclusion section (Page 14, Lines 467–471) to reflect this broader applicability beyond healthy individuals. These revisions aim to highlight the translational potential of our findings and reinforce the relevance of Bot Fit in both clinical and fitness domains.**

***Page 13, Lines 427–449:***

*Beyond its application to strength assessment in healthy adults, the Bot Fit system may also hold promise for clinical and rehabilitation settings. Although this study focused on young, healthy individuals, the wearable robotic platform could potentially be adapted to support individuals with reduced mobility or those undergoing physical therapy. Future studies involving older adults or clinical populations will be necessary to evaluate the system’s usability and effectiveness in these contexts.*

***Page 14, Lines 468–472:***

*Future research should expand the sample size and incorporate additional data sources, such as sEMG and motor signal analysis, to improve model validity and robustness. Through continued development, Bot Fit may serve as a valuable platform for both fitness evaluation and rehabilitation support, contributing to more efficient and accessible muscular strength assessment.*

**6. PLOS authors have the option to publish the peer review history of their article (**[**what does this mean?**](https://journals.plos.org/plosone/s/editorial-and-peer-review-process#loc-peer-review-history)**). If published, this will include your full peer review and any attached files.
If you choose “no”, your identity will remain anonymous but your review may still be made public.
Do you want your identity to be public for this peer review? For information about this choice, including consent withdrawal, please see our** [**Privacy Policy**](https://www.plos.org/privacy-policy)**.**

**Reviewer #1: Yes: Rodrigo Assunção de Oliveira**

**Reviewer #2: Yes: Diana Ciubotariu**

**We would like to express our sincere gratitude to both Reviewer #1 and Reviewer #2 for their thoughtful, constructive, and encouraging comments. Your insights have been invaluable in improving the clarity, depth, and relevance of our manuscript. We deeply appreciate the time and expertise you dedicated to reviewing our work.**
